# Supplementary material for: Mitochondrial respiratory function is preserved under cysteine starvation via glutathione catabolism in NSCLC
Source: Nat Commun. 2024 May 18;15:4244. doi: 10.1038/s41467-024-48695-2 (PMC11102494; doi:10.1038/s41467-024-48695-2)
Supplement: Supplementary file 1 — Supplementary Information [file 41467_2024_48695_MOESM1_ESM.pdf]

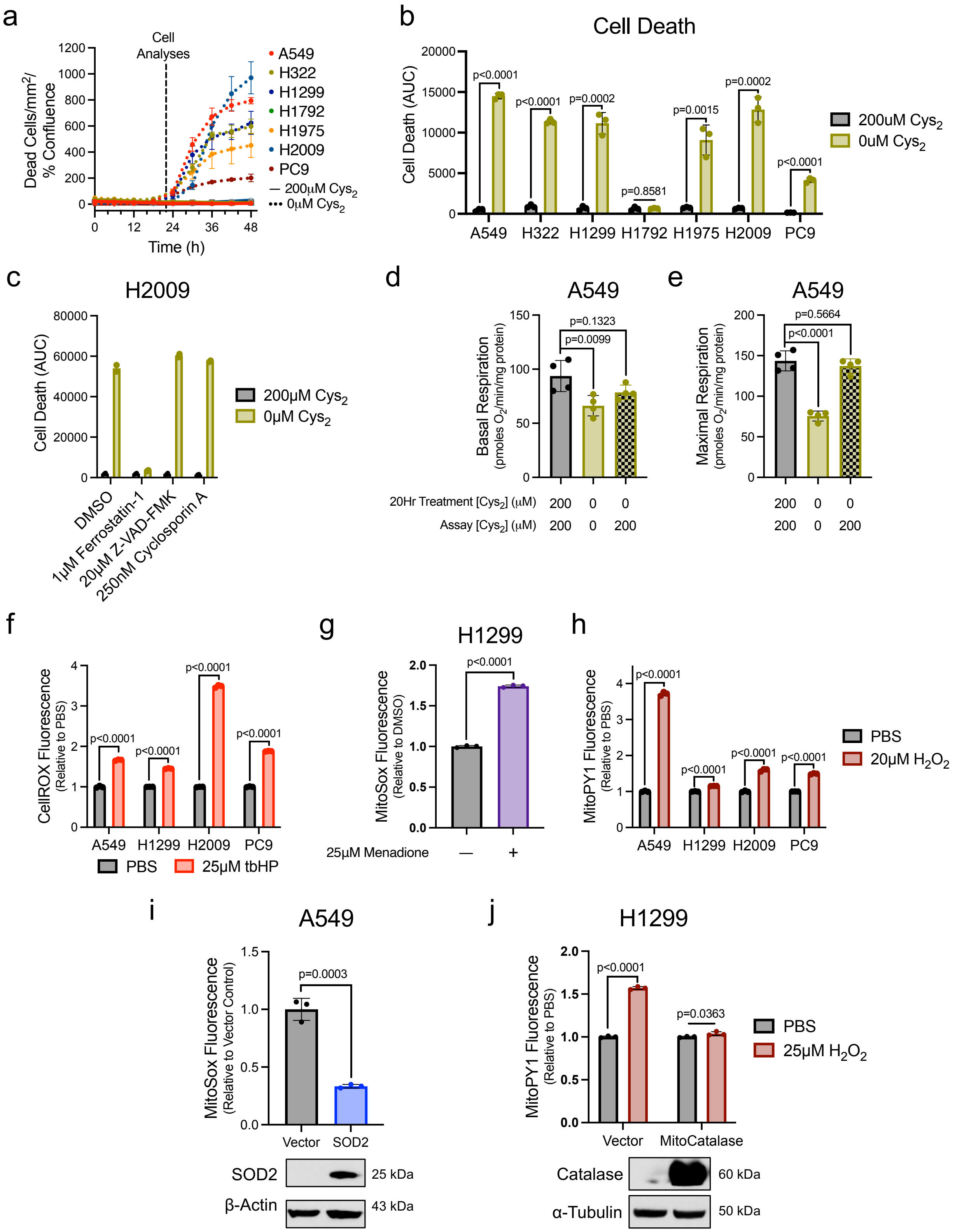

a
Cell Analyses
Dead Cells/mm²/ % Confluence
Time (h)
A549
H322
H1299
H1792
H1975
H2009
PC9
200µM Cys2
0µM Cys2
b
Cell Death
Cell Death (AUC)
p<0.0001
p<0.0001
p=0.0002
p=0.8581
p=0.0015
p=0.0002
p<0.0001
200uM Cys2
0uM Cys2
c
H2009
Cell Death (AUC)
200µM Cys2
0µM Cys2
DMSO
1µM Ferrostatin-1
20µM Z-VAD-FMK
250nM Cyclosporin A
d
A549
Basal Respiration (pmoles O2/min/mg protein)
p=0.1323
p=0.0099
20Hr Treatment [Cys2] (µM)
Assay [Cys2] (µM)
e
A549
Maximal Respiration (pmoles O2/min/mg protein)
p=0.5664
p<0.0001
f
CellROX Fluorescence (Relative to PBS)
PBS
25µM tbHP
g
H1299
MitoSox Fluorescence (Relative to DMSO)
p<0.0001
25µM Menadione
h
MitoPY1 Fluorescence (Relative to PBS)
PBS
20µM H2O2
i
A549
MitoSox Fluorescence (Relative to Vector Control)
p=0.0003
Vector
SOD2
SOD2
β-Actin
25 kDa
43 kDa
j
H1299
MitoPY1 Fluorescence (Relative to PBS)
p<0.0001
p=0.0363
PBS
25µM H2O2
Vector
MitoCatalase
Catalase
α-Tubulin
60 kDa
50 kDa

Supplementary Figure 1: **NSCLC Viability is Susceptible to Cys_2 Starvation**. **a**, Plot of the accumulation of dead NSCLC cells over time in cystine replete (solid) or starved (dashed) conditions (n=3 biologically independent samples per condition). **b**, Measurement of NSCLC cell death over 48h in the presence or absence of cystine. Data represented as area under the curve (AUC) of the plots depicted in **a** (n=3 biologically independent samples per condition). **c**, Measurement of cell death over 48h in H2009 cells fed/starved of cystine and treated with the indicated inhibitors of ferroptosis (Ferrostatin-1), apoptosis (Z-VAD-FMK), or necroptosis (Cyclosporin A) (n=2 biologically independent samples per condition). **d** and **e**, A549 cells were either fed or starved of 200μM cystine (Cys_2) for 20Hr and then subject to Seahorse MitoStress Test Analysis of **d**, basal respiration, or **e**, maximal respiration. Cys_2-starved cells were either assayed in the presence or absence of Cys_2 (n=4 biologically independent samples per condition). **f**, Relative CellROX Green fluorescence in NSCLC cells treated with PBS or 25μM tert-butyl hydroperoxide (tbHP) for 30 minutes (n=3 biologically independent samples per condition). **g**, Relative MitoSOX Red fluorescence of H1299 cells treated with DMSO or 25μM menadione-containing media for 30 minutes (n=3 biologically independent samples per condition). **h**, Relative MitoPY1 fluorescence in NSCLC cells treated with PBS or 20μM H_2O_2 for 30 minutes (n=3 biologically independent samples per condition). **i**, Relative basal MitoSOX Red fluorescence in control or SOD2-overexpressing A549 cells (n=3 biologically independent samples per condition). **j**, Relative MitoPY1 fluorescence in vector-control or MitoCatalase infected H1299 cells challenged with 25μM H_2O_2 for 30 minutes (n=3 biologically independent samples per condition). Data represent mean values ± s.d. For **b** and **f-i**, P values were calculated using a two-tailed unpaired Student's

t-test. For **c and j**, P values were calculated using a two-way ANOVA. For **d and e**, P values were calculated using a one-way ANOVA. All data are representative of at least 3 experimental replicates. For **a-j**, source data provided as a Source Data file.

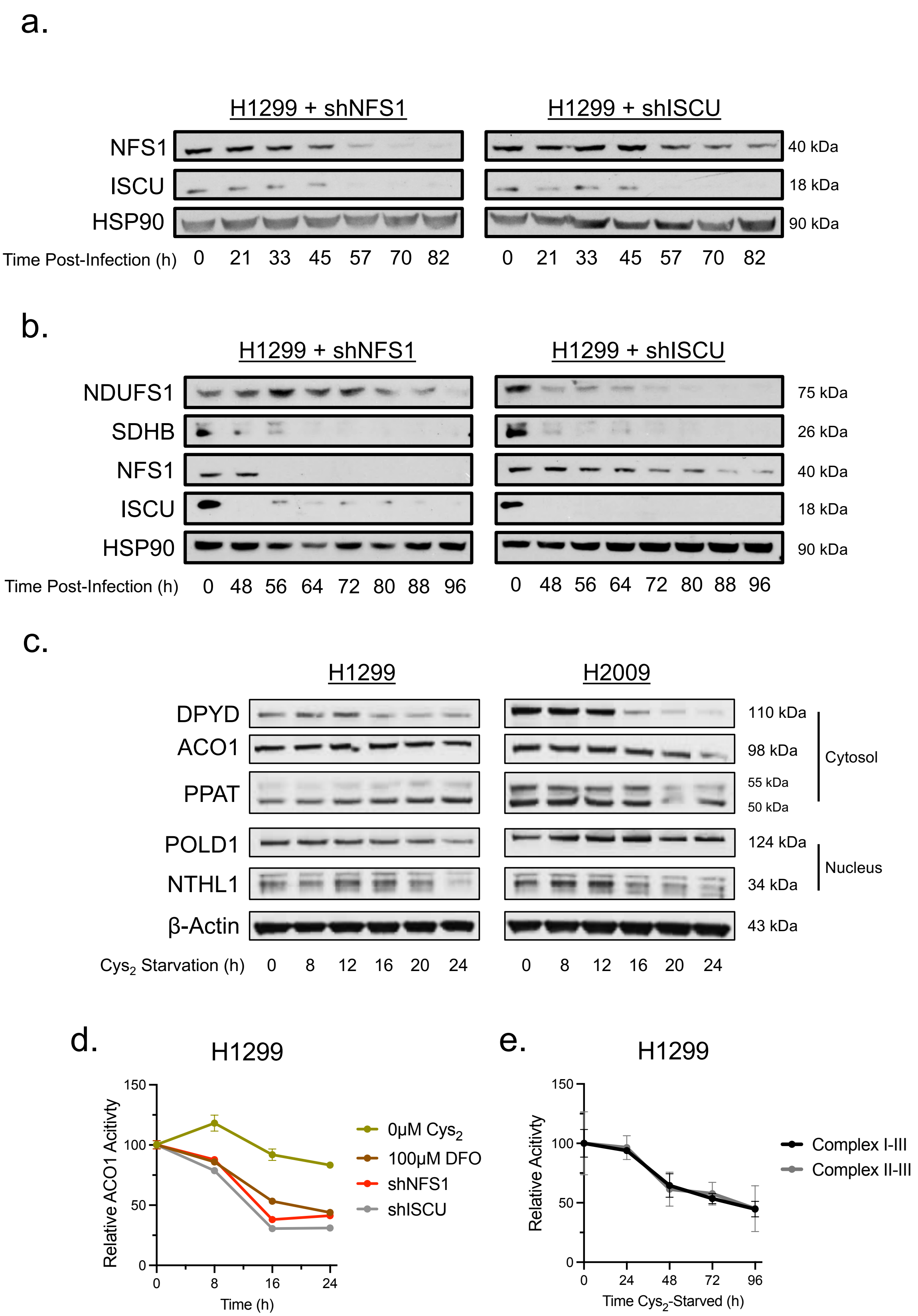
a.
H1299 + shNFS1
H1299 + shISCU
NFS1
ISCU
HSP90
40 kDa
18 kDa
90 kDa
Time Post-Infection (h)
0 21 33 45 57 70 82
0 21 33 45 57 70 82
b.
H1299 + shNFS1
H1299 + shISCU
NDUFS1
SDHB
NFS1
ISCU
HSP90
75 kDa
26 kDa
40 kDa
18 kDa
90 kDa
Time Post-Infection (h)
0 48 56 64 72 80 88 96
0 48 56 64 72 80 88 96
c.
H1299
H2009
DPYD
ACO1
PPAT
POLD1
NTHL1
β-Actin
110 kDa
98 kDa
55 kDa
50 kDa
124 kDa
34 kDa
43 kDa
Cytosol
Nucleus
Cys_2 Starvation (h)
0 8 12 16 20 24
0 8 12 16 20 24
d.
H1299
Relative ACO1 Acitivty
150
100
50
0
0 8 16 24
Time (h)
0μM Cys_2
100μM DFO
shNFS1
shISCU
e.
H1299
Relative Acitivty
150
100
50
0
0 24 48 72 96
Time Cys_2-Starved (h)
Complex I-III
Complex II-III

Supplementary Figure 2: **Extramitochondrial Fe-S Proteins Exhibit Varied Stability in Response to Cystine Starvation**. **a**, Immunoblot analysis of the kinetics of shRNA-mediated knockdown of NFS1 and ISCU in H1299 cells subject to lentiviral infection. **b**, Immunoblot analysis of Fe-S protein expression (NDUFS1, SDHB) upon shRNA-mediated knockdown of NFS1 and ISCU in H1299 cells subject to lentiviral infection. **c**, Immunoblot analysis of the expression of extramitochondrial Fe-S proteins (DPYD, ACO1, PPAT, POLD1, NTHL1) and β-Actin in H1299 and H2009 cells following treatment with cystine-deficient media supplemented with 1μM ferrostatin-1 for the indicated time points. **d**, ACO1 activity in cytosolic lysates of H1299 cells following treatment with cystine starvation, DFO, or upon knockdown of NFS1 or ISCU at the indicated time points (n=3 biologically independent samples per condition); data points are representative of ACO1 activity of each treatment group relative to the activity of that group at time 0h. **e**, ETC supercomplex activites in permeabilized H1299 cells following treatment with cystine-deficient media supplemented with 1μM ferrostatin-1 for the indicated time points (n=3 biologically independent samples per condition). Data points are representative of the activity of each supercomplex relative to cystine-replate conditions (time 0h). Data represent mean values ± s.d, and are representative of at least 3 experimental replicates. For **a-e**, source data are provided as a Source Data file.

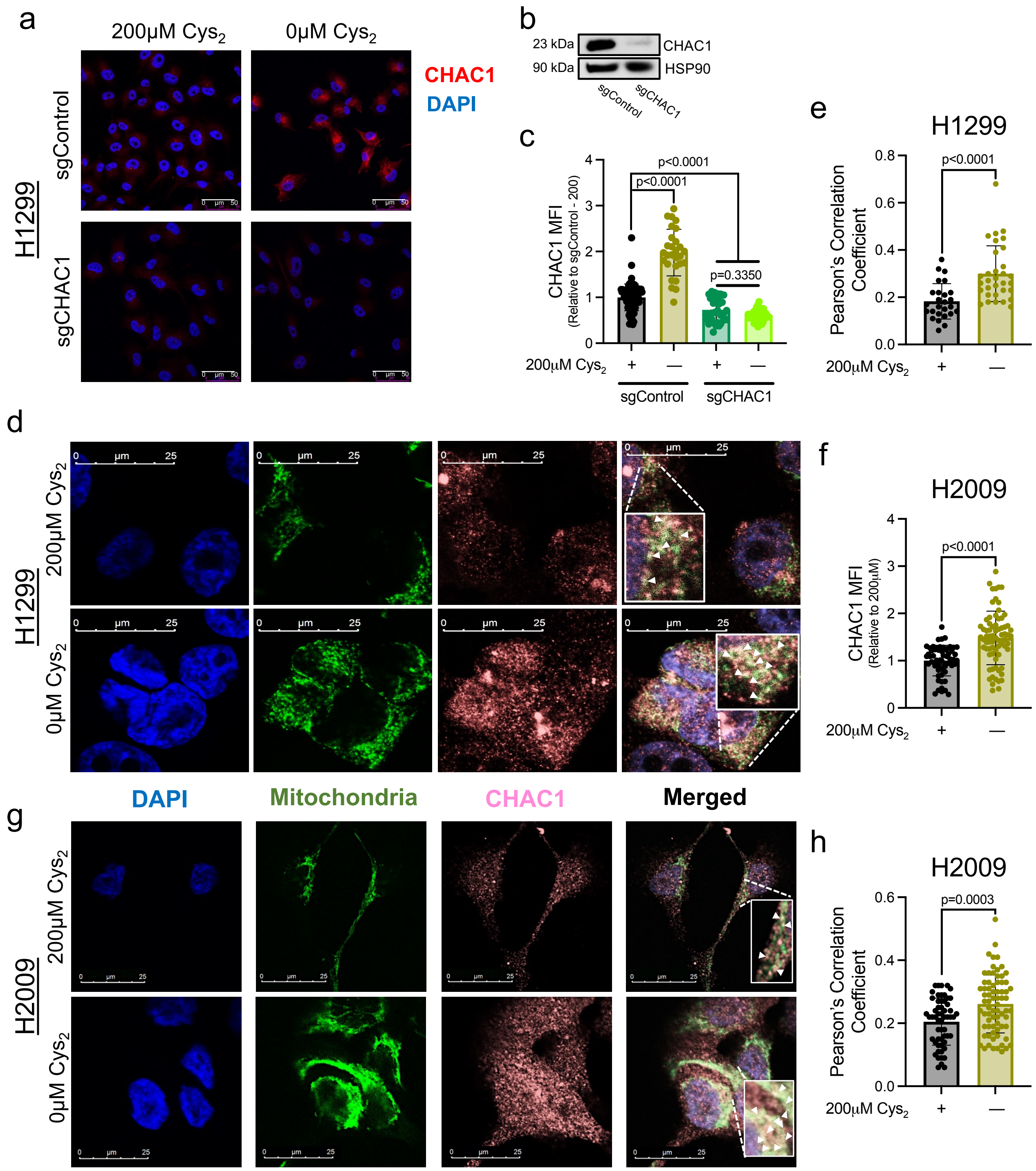

Supplementary Figure 3: CHAC1 is Diffusely Expressed Throughout the Cell, Including the Mitochondria. a, Representative CHAC1 immunofluorescence images of fixed CHAC1 expressing or deficient H1299 cells subject to 20Hr culturing in cystine-

replete or starved conditions. Following treatment, cells were fixed and sequentially subjected to a specific primary antibody towards CHAC1, an Alexa Fluor-647 conjugated secondary (red), and DAPI (blue) prior to analysis. **b**, Immunoblot analysis of CHAC1 and HSP90 expression of the H1299 cells depicted in panel **a**. **c**, Quantification of mean CHAC1 fluorescence intensity in fixed CHAC1 expressing or deficient H1299 cells subject to a 20h incubation in cystine-replete deficient media (n=79 cells for sgControl-200μM Cys_2, n=28 cells for sgControl-0μM Cys_2, n=33 cells for sgCHAC1-200μM Cys_2, and n=45 cells for sgControl-0μM Cys_2). **d**, Representative immunofluorescent images of Mito-Grx1-roGFP2 expressing H1299 cells fed or starved of cystine for 20h. **e**, Pearson correlation coefficient analysis of CHAC1 (magenta) colocalization with mitochondria (green) in H1299 cells subject to culturing in cystine replete (n=25 cells) or starved conditions (n=32 cells) for 20h in at least 3 separate fields of view. **f**, Quantification of mean CHAC1 fluorescence intensity in fixed H2009 cells subject to a 20h incubation in cystine-replete (n=57 cells) or deficient media (n=72 cells). **g**, Representative immunofluorescent images of Mito-Grx1-roGFP2 expressing H1299 cells fed or starved of cystine for 20h. For **c** and **f**, data representative of the fluorescence intensity of discrete cells in at least 3 separate fields of view. **h**, Pearson correlation coefficient analysis of CHAC1 (magenta) colocalization with mitochondria (green) in H2009 cells subject to culturing in cystine replete (n=57 cells) or starved conditions (n=72 cells) for 20h in 3 separate fields of view. For **d** and **h**, following treatment, Mito-Grx1-roGFP2-expressing (green) cells were fixed and sequentially subjected to a specific primary antibody towards CHAC1, an Alexa Fluor-647 conjugated secondary (magenta), and DAPI (blue) prior to analysis. White arrows indicate colocalization (yellow) of CHAC1 and Mito-Grx1-roGFP2

signals. For **e** and **g**, data represent the Pearson correlation coefficients of the physical overlap in red and green pixels in merged images of red (Alexa Fluor 647) and green (Mito-Grx1-roGFP) fluorescence channels; each data point represents a discrete cell. For **c**, data represent mean values ± s.d. For **c**, P values were calculated using a one-way ANOVA. For **e, f,** and **h**, P values were calculated using a two-tailed unpaired Student's *t*-test**.** For **b**, **c**, **e**, **f**, and **h**, source data provided as a Source Data file.

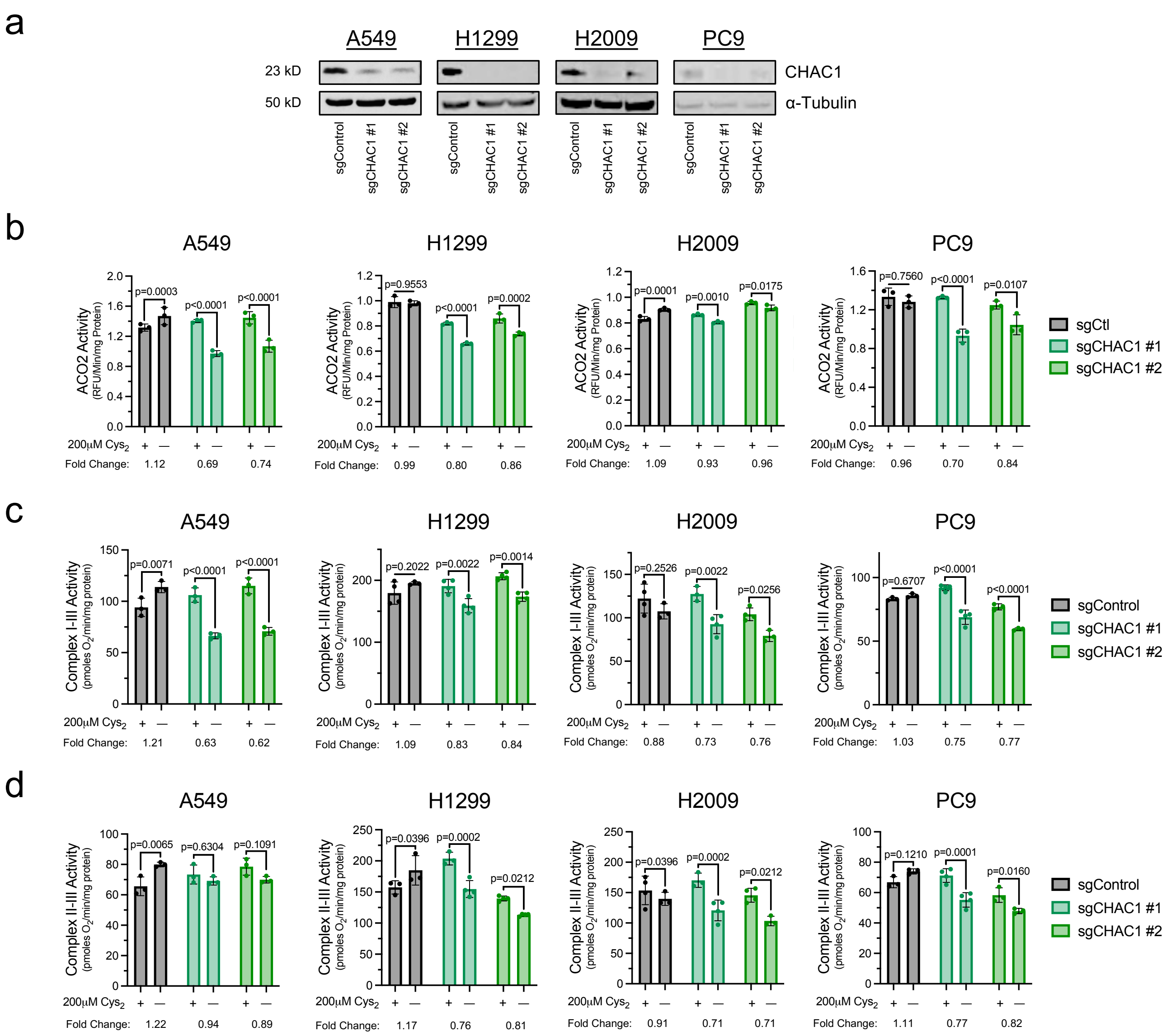

Supplementary Figure 4: **CHAC1 Knockout Reduces Fe-S Protein Function Only in the Absence of Cys₂.** **a**, immunoblot analysis of CHAC1 and α-Tubulin expression in NSCLC cells subject to CRISPR/Cas9-mediated knockout of CHAC1. **b**, ACO2 activities in CHAC1-deficient NSCLC cells subject to either cystine replete or starved conditions (n=3 biologically independent samples per condition). **c** and **d**, ETC supercomplex activities in permeabilized CHAC1-deficient cells stimulated with their associated substrates following culture in the presence of absence of cystine (n =3 biologically

independent samples per condition, except when n=4 for H1299+sgControl-200μM, H1299+sgCHAC1 #1, H1299+sgCHAC1 #2, H2009+sgControl-200μM, H2009+sgCHAC1 #1-0μM, H2009+sgCHAC1 #2-200μM, PC9+sgCHAC1 #1 groups). Data represent mean values ± s.d. For **b-d,** data are representative of at least 3 experimental replicates and P values were calculated using a two-way ANOVA. For **a-d**, source data are provided as a Source Data file.

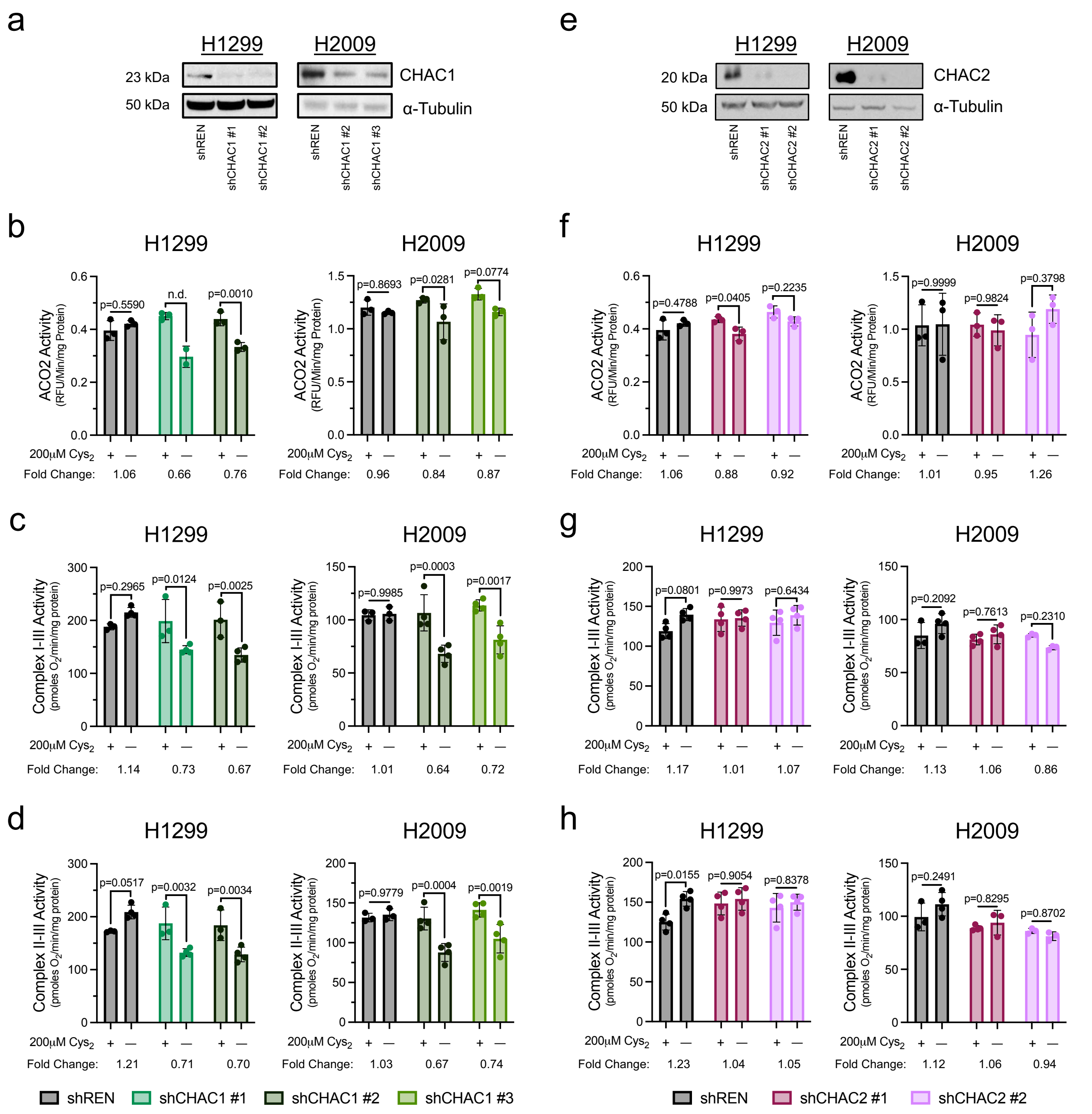

Supplementary Figure 5: **CHAC2 Loss Does Not Influence Fe-S Protein Function. a**, immunoblot analysis of CHAC1 and α-Tubulin expression in H1299 and H2009 cells subject to 5-days of 0.2μg/mL doxycycline treatment to induce shRNA-mediated knockdown of CHAC1. **b**, ACO2 activities in H1299 and H2009 CHAC1-knockdown cells subject to either cystine replete or starved conditions (n=3 per condition, except for when

n=2 for H1299+shCHAC1 #1-0μM). **c** and **d**, ETC supercomplex activities in permeabilized CHAC1-knockdown cells stimulated with **c**, 10mM pyruvate and 1mM malate or **d**, 10mM succinate following culture in the presence of absence of cystine (n=3 biologically independent samples per condition, except when n=4 for H1299+shREN-0μM, H1299+shCHAC1 #1-0μM, H1299+shCHAC1 #2-0μM, H2009+shCHAC1 #1, and H2009+shCHAC1 #2 groups). **e**, Immunoblot analysis of CHAC2 and α-Tubulin expression in H1299 and H2009 cells subject to 5-days of 0.2μg/mL doxycycline treatment to induce shRNA-mediated knockdown of CHAC2. **f**, ACO2 activities in H1299 and H2009 CHAC2-knockdown cells cultured in the presence or absence of cystine (n=3 biologically independent samples per condition). **g** and **h**, ETC supercomplex activities in permeabilized CHAC2-knockdown cells stimulated with their associated substrates following culture in cystine replete or starved conditions (n=4 biologically independent samples per condition, except for when n=3 for H2009+shREN-200μM, H2009+shCHAC2 #1-0μM, H2009+shCHAC2 #2 groups). For **b-d** and **f-h**, cells were treated with 0.2μg/mL doxycycline for 5 days prior to analysis and cultured in the indicated [cystine] for the final 20h. Data represent mean values ± s.d; n.d., not determined. For **b-d** and **f-h**, data are representative of at least 3 experimental replicates and P values were calculated using a two-way ANOVA. For **b-d** and **f-h**, source data are provided as a Source Data file.

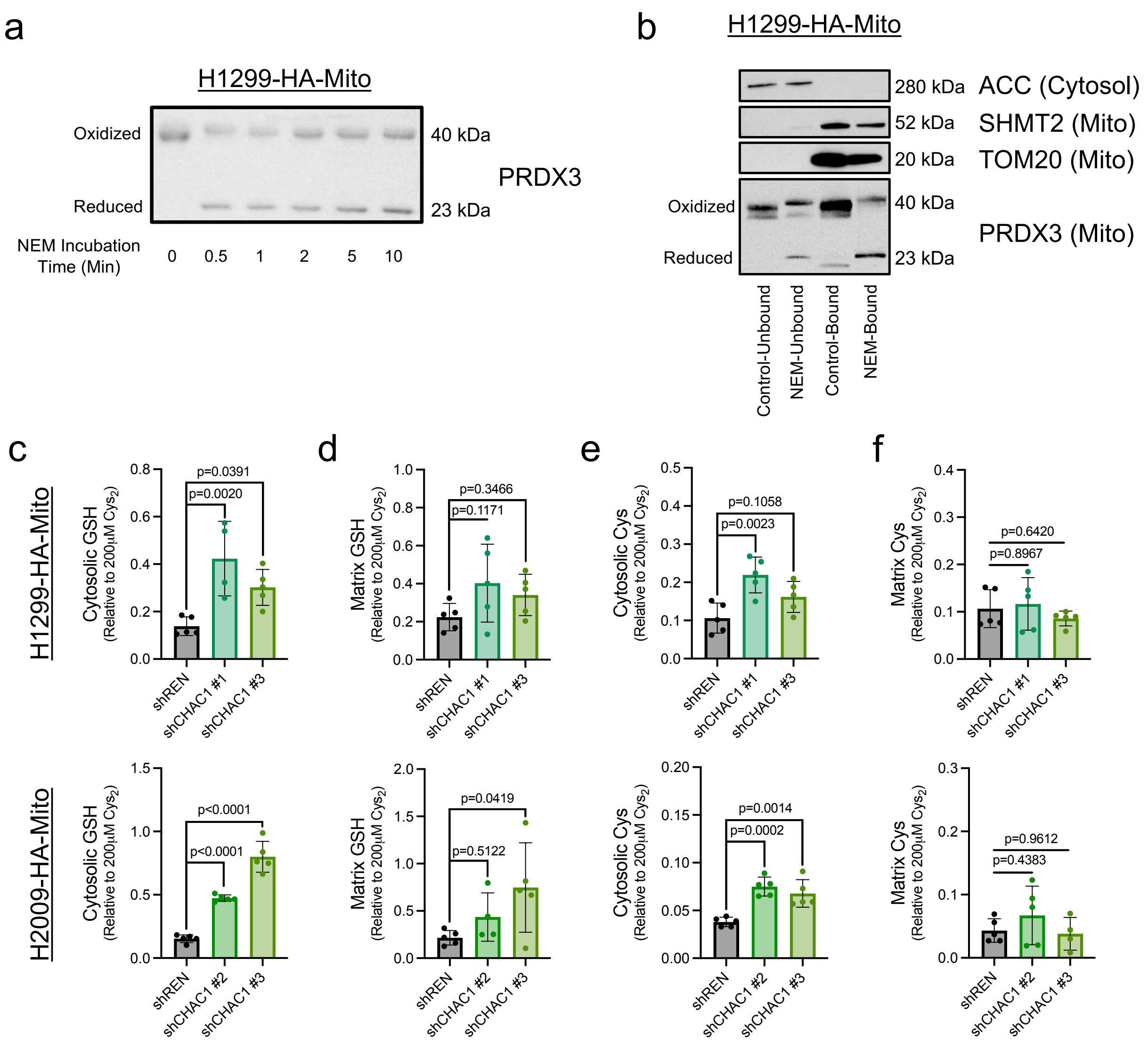

Supplementary Figure 6: CHAC1 Modulates GSH Availability Across Compartments Under Cysteine Restriction. a, Redox immunoblot determination of reduced PRDX3 stabilization in H1299-HA-Mito cells treated with 25mM NEM for up to 10min prior to mitochondrial immunoprecipitation. **b**, Immunoblot analysis of ACC, PRDX3, SHMT2, TOM20 expression in H1299-HA-Mito cells following mitochondrial immunoprecipitation ± a 1 minute incubation with 25mM NEM prior to mitochondrial isolation. **c-f**, H1299 and H2009-HA-Mito cells were treated with 0.2µg/mL doxycycline

for 5 days to induce shRNA knockdown of CHAC1 and then subject to cystine starvation for 12h. The pool sizes of **c**, cytosolic GSH (n=5 biologically independent samples, except when n=4 for H1299+shCHAC1 #1), **d**, matrix GSH (n=5 biologically independent samples, except when n=4 for H2009+shCHAC1 #1), **e**, cytosolic cysteine (n=5 biologically independent samples), and **f**, matrix cysteine (n=5 biologically independent samples, except when n=4 for H2009+shCHAC1 #2) were then determined relative to cystine replete conditions. Data represent mean values ± s.d. For **c-f**, P values were calculated with a one-way ANOVA. For **a-f**, source data are provided as a Source Data file.

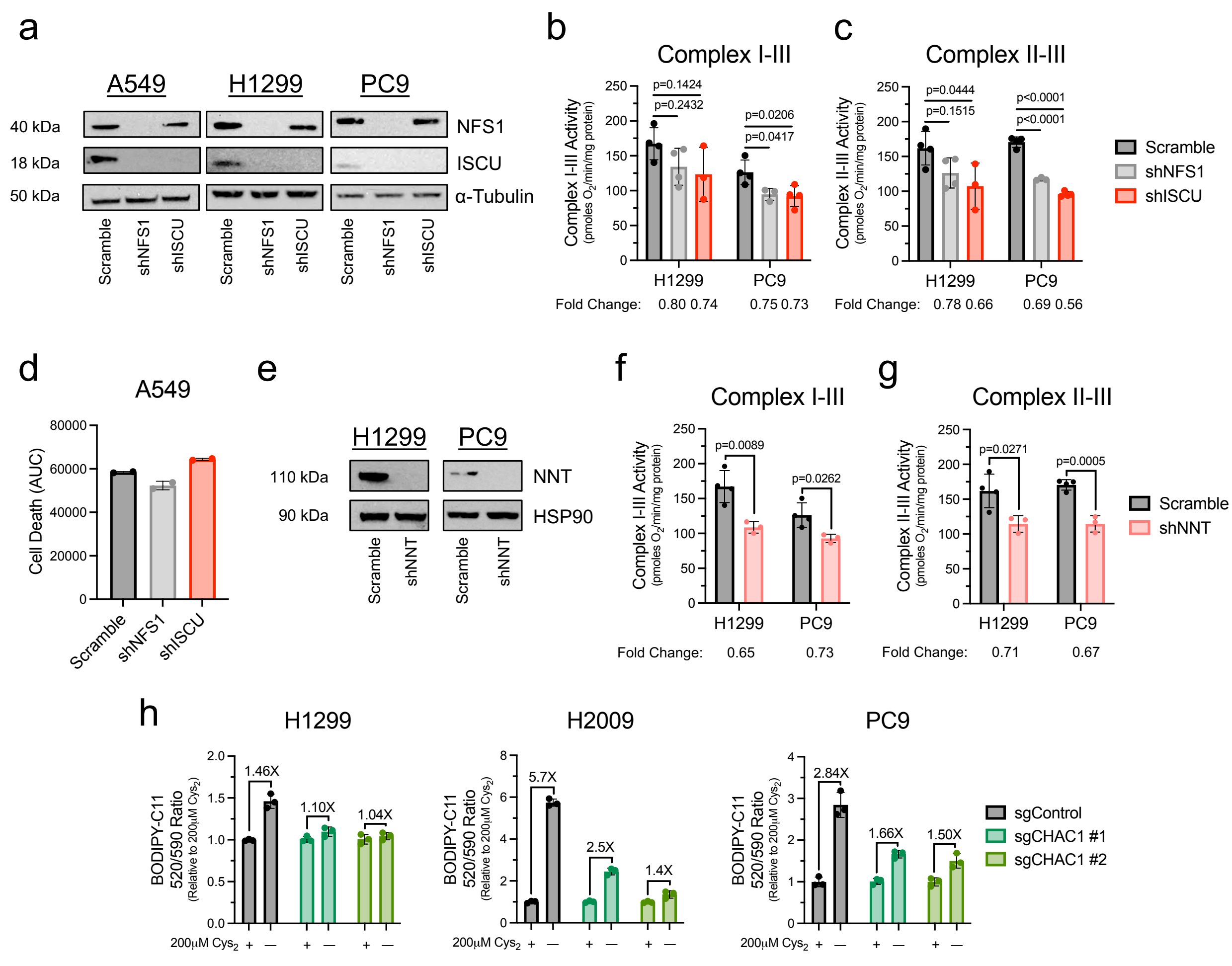

Supplementary Figure 7: **Fe-S Protein Function is Diminished Upon Disruption of Fe-S Cluster Homeostasis**. **a**, Immunoblot analysis of NFS1, ISCU, and α-Tubulin expression in A549, H1299, and PC9 cells 4d post-infection with scramble, shNFS1, or shISCU lentivirus. **b** and **c**, ETC supercomplex activities in permeabilized H1299 and PC9 cells stimulated with **b**, 10mM pyruvate and 1mM malate or **c**, 10mM succinate 4d post-infection with scramble, shNFS1, or shISCU lentivirus (n =4 biologically independent samples per condition, except when n=3 for H1299+shISCU and H2009+shNFS1). **d**, Quantification of cell death over 48h of cystine starvation in A549 cells following the disruption of Fe-S cluster synthesis through knockdown of NFS1 and ISCU (n=2 per condition). **e**, Immunoblot analysis of NNT and HSP90 expression in H1299, and PC9

cells 4d post-infection with scramble or shNNT lentivirus. **f** and **g**, ETC supercomplex activities in permeabilized H1299 and PC9 cells stimulated with their associated substrates 4d post-infection with scramble or shNNT lentivirus (n=4 biologically independent samples for scramble infected cells and n=3 for shNNT infected cells). **h**, Relative BODIPY-C11 fluorescence ratio of CHAC1-deficient H1299, H2009, and PC9 cells cultured in the presence or absence of cystine for ≥20h as an indicator of the extent of cellular lipid peroxidation (n=3 biologically independent samples per condition). Data represent mean values ± s.d.. For **b** and **c,** P values were calculated using a one-way ANOVA. For **f** and **g**, P values were calculated using a two-tailed unpaired Student's *t*-test**.** Data are representative of at least 3 experimental replicates. For **a-h**, source data are provided as a Source Data file.

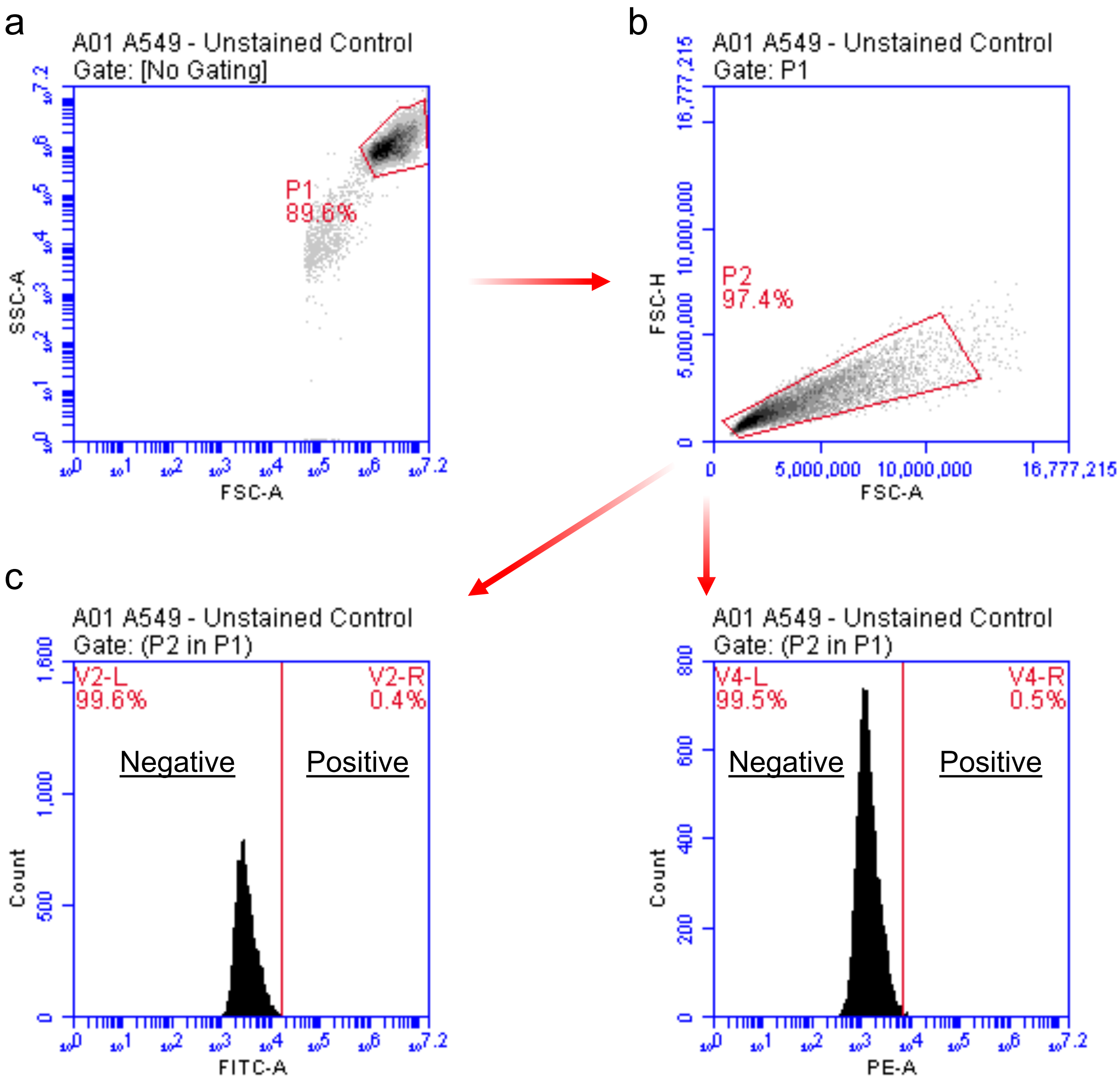

Supplementary Figure 8: Representative Gating Scheme for Flow Cytometry Analyses of ROS. **a**, For all analyses, live NSCLC cells were identified with an initial FSC/SSC gating scheme (FSC > 500,000; SSC > 500,000). **b**, Cell doublets were then excluded through subsequent gating based on forward scatter-height and forward scatter-area (FSC-H v. FSC-A). **c**, Cells positively stained for the respective fluorescent ROS indicator were identified based on gating established by determining the FITC and/or PE

autofluorescence of unstained control cells. Cells present to the left of these gates on the corresponding histograms were considered unstained or negative, where those to the right of these gates were considered positively stained and included for analysis.

Supplementary Table 1

Oligonucleotide	Source	Identifier
Guide RNA for lentiCRISPR-V2-CHAC1 #1; Forward: 5’- caccgGAAGTCGGGCCTCCACACCA-3’; Reverse: 5’- aaacTGGTGTGGAGGCCCGACTTCc-3’	Birsoy et al., 2015	N/A
Guide RNA for lentiCRISPR-V2-CHAC1 #2; Forward: 5’- caccgGGTACGGCTCCCTGGTGTGG-3’; Reverse: 5’- aaacCCACACCAGGGAGCCGTACCc-3’	Birsoy et al., 2015	N/A
Antisense Hairpin Sequence for shCHAC1 #1 – 5’-TCAAGTAGTAAGTCAGTGTCAA-3’	This Study	N/A
Antisense Hairpin Sequence for shCHAC1 #2 – 5’-GCCTCTTACCCACTTGGTTGTT-3’	Yang et al., 2011	TRCN0000157739
Antisense Hairpin Sequence for shCHAC1 #3 – 5’-ACCAAGGAGGTCACCTTCTATC-3’	Yang et al., 2011	TRCN0000275981
Antisense Hairpin Sequence for shCHAC2 #1 – 5’-TAGAATTTCAAAATAGTATTGG-3’	This Study	N/A
Antisense Hairpin Sequence for shCHAC2 #2 – 5’-GCCTCTTACCCACTTGGTTGTT-3’	This Study	N/A
Hairpin Sequence for Scramble – 5’-CAACAAGATGAAGAGCACCAACTCGAGTTGGTGCTCTTCATCTTGTTG-3’	Millipore Sigma	SHC002
Antisense Hairpin Sequence for shNFS1 – 5’-CAGTTCCAGAAAGGTATATTT-3’	Addgene	Cat #: 102963
Antisense Hairpin Sequence for shISCU – 5’-GTCCCTTGACAAGACATCTAA-3’	Addgene	Cat #: 102972
Hairpin Sequence for shNNT – 5’-AATAATGCTATTAGCTTCTCGTCAAGAGCGAGAAGCTAATAGCATTATT-3’	Open Biosystems	TRCN0000028507
