# Supplementary material for: Mitochondrial respiratory function is preserved under cysteine starvation via glutathione catabolism in NSCLC
Source: Nat Commun. 2024 May 18;15:4244. doi: 10.1038/s41467-024-48695-2 (PMC11102494; doi:10.1038/s41467-024-48695-2)
Supplement: Supplementary file 3 — Reporting Summary [file 41467_2024_48695_MOESM3_ESM.pdf]

nature portfolio

Corresponding author(s): Gina M. DeNicola, Nathan P. Ward

Last updated by author(s): Apr 18, 2024

Reporting Summary

Nature Portfolio wishes to improve the reproducibility of the work that we publish. This form provides structure for consistency and transparency in reporting. For further information on Nature Portfolio policies, see our Editorial Policies and the Editorial Policy Checklist.

Statistics

For all statistical analyses, confirm that the following items are present in the figure legend, table legend, main text, or Methods section.

n/a	Confirmed	
☐	☒	The exact sample size (*n*) for each experimental group/condition, given as a discrete number and unit of measurement
☐	☒	A statement on whether measurements were taken from distinct samples or whether the same sample was measured repeatedly
☐	☒	The statistical test(s) used AND whether they are one- or two-sided<br>*Only common tests should be described solely by name; describe more complex techniques in the Methods section.*
☒	☐	A description of all covariates tested
☐	☒	A description of any assumptions or corrections, such as tests of normality and adjustment for multiple comparisons
☐	☒	A full description of the statistical parameters including central tendency (e.g. means) or other basic estimates (e.g. regression coefficient) AND variation (e.g. standard deviation) or associated estimates of uncertainty (e.g. confidence intervals)
☐	☒	For null hypothesis testing, the test statistic (e.g. *F*, *t*, *r*) with confidence intervals, effect sizes, degrees of freedom and *P* value noted<br>*Give P values as exact values whenever suitable.*
☒	☐	For Bayesian analysis, information on the choice of priors and Markov chain Monte Carlo settings
☒	☐	For hierarchical and complex designs, identification of the appropriate level for tests and full reporting of outcomes
☐	☒	Estimates of effect sizes (e.g. Cohen's *d*, Pearson's *r*), indicating how they were calculated

Our web collection on statistics for biologists contains articles on many of the points above.

Software and code

Policy information about availability of computer code

Data collection	CYTENA CellCyte Studio software (Version 2.7; Freiburg, Germany) was used to acquire bright-field and fluorescent images for analyses of cell death. Seahorse Wave software (Version 2.4.3; Agilent Technologies, Santa Clara, CA, USA) was used in the collection of oxygen consumption data for seahorse experiments. BD CSampler Plus software (Version 1.0.34.1; BD Biosciences, Franklin Lakes, NJ, USA) was used to collect raw flow cytometry data. Leica Application Suite X software (Version 5.1; Leica Microsystems, Deerfield, IL, USA) was used to acquire immunofluorescence images.
Data analysis	GraphPad Prism 9.4.1 (458) was used for statistical analysis.<br>LC-MS metabolite data was analyzed with El Maven v0.3.1.<br>Mean fluorescence intensity and signal colocalization of immunofluorescence images was analyzed with Fiji.

For manuscripts utilizing custom algorithms or software that are central to the research but not yet described in published literature, software must be made available to editors and reviewers. We strongly encourage code deposition in a community repository (e.g. GitHub). See the Nature Portfolio guidelines for submitting code & software for further information.

March 2021

Data

Policy information about availability of data

All manuscripts must include a data availability statement. This statement should provide the following information, where applicable:

- Accession codes, unique identifiers, or web links for publicly available datasets
- A description of any restrictions on data availability
- For clinical datasets or third party data, please ensure that the statement adheres to our policy

All data supporting the findings of this study are provided within the figures and Source Data files.

Human research participants

Policy information about studies involving human research participants and Sex and Gender in Research.

Reporting on sex and gender	N/A
Population characteristics	N/A
Recruitment	N/A
Ethics oversight	N/A

Note that full information on the approval of the study protocol must also be provided in the manuscript.

Field-specific reporting

Please select the one below that is the best fit for your research. If you are not sure, read the appropriate sections before making your selection.

[x] Life sciences [ ] Behavioural & social sciences [ ] Ecological, evolutionary & environmental sciences

For a reference copy of the document with all sections, see nature.com/documents/nr-reporting-summary-flat.pdf

Life sciences study design

All studies must disclose on these points even when the disclosure is negative.

Sample size	No statistical methods were used to determine sample size. Biological triplicates were used for most experiments based on manufacturers recommendations for use of commercially available reagents/assays or based on our previously published work (doi: 10.1084/jem.20191689). Seahorse analyses included quadruplicate replicates as recommended by the manufacturer, and LC-MS analyses of cytosolic and matrix pools of cysteine and glutathione employed up to 5 replicates due to high variability.
Data exclusions	Biological replicates for Seahorse analyses were excluded when OCR values were recorded by the instrument as negative values; this applies to Figure panels 3c, 3d, and Extended Data Figure panels 4c, 4d, 5c, 5d, 5g, 5h, 7b, 7c.<br><br>Outlier values were identified using GraphPad Prism 9.4.1 software, which applied Grubb's test (extreme studentized deviant method) to data points in each group. 1 outlier each were excluded from sample groups in panels Extended Data Figure 6c (H1299+shCHAC1 #1 and H2009 +shREN), 6d (H2009+shCHAC1 #2), and 6f (H2009+shCHAC1 #3).<br><br>No experimental groups or cell lines were wholly excluded from any analysis.<br><br>Exclusion criteria were not predetermined and were part of the overall experimental quality control procedures.
Replication	For LC-MS analyses of the cytosolic and matrix pools of cysteine and glutathione, data are representative of experiments conducted with 3-5 biological replicates (distinct cell cultures). The experiments associated with Supplementary Figure 6 were repeated multiple times for protocol optimization with similar results obtained each time. For all other experiments, at least 3 biological replicates were included for each group when possible and each experiment repeated three times with similar outcomes.
Randomization	Cell line samples were randomly assigned to treatment groups. For LC-MS analyses, cytosolic and mitochondrial samples were analyzed in a random order.
Blinding	Investigators were not blinded during data collection or analysis.

Reporting for specific materials, systems and methods

We require information from authors about some types of materials, experimental systems and methods used in many studies. Here, indicate whether each material, system or method listed is relevant to your study. If you are not sure if a list item applies to your research, read the appropriate section before selecting a response.

Materials & experimental systems

n/a	Involved in the study
☐	☒ Antibodies
☐	☒ Eukaryotic cell lines
☒	☐ Palaeontology and archaeology
☒	☐ Animals and other organisms
☒	☐ Clinical data
☒	☐ Dual use research of concern

Methods

n/a	Involved in the study
☒	☐ ChIP-seq
☐	☒ Flow cytometry
☒	☐ MRI-based neuroimaging

Antibodies

Antibodies used

ACC - Cell Signaling Technology, 3662, Lot #8
ACO1 - GeneTex, GTX128976, Lot #41451
ACO2 - GeneTex, GTX109736, Lot #40030
AIF - Cell Signaling Technology, 4642, Lot #3
ATF4 - Cell Signaling Technology, 11815, Lot #2
Beta-Actin - Thermo Fisher Scientific, AM4302, Lot #01171591
Catalase - Cell Signaling Technology, 12980, Lot #2
CHAC1 - Proteintech, 15207-1-AP, Lot #00015028
CHAC2 - GeneTex, GTX128819, Lot #41423
CS - Cell Signaling Technology, 14309, Lot #2
DLST - Cell Signaling Technology, 11954, Lot #1
DPYD - Cell Signaling Technology, 4654, Lot #2
ERp44 - Cell Signaling Technology, 3798, Lot #2
FECH - Santa Cruz Biotechnology, sc-377377, Lot #A1223
Grp94 - Cell Signaling Technology, 2104, Lot #2
HSP60 - Cell Signaling Technology, 4870, Lot #2
HSP90 - Cell Signaling Technology, 4874, Lot #6
ISCU - Santa Cruz Biotechnology, sc-373694, Lot #J1717
Lamin B2 - Cell Signaling Technology, 12255, Lot #1
LIAS - Proteintech, 11577-1-AP, Lot #00114850
Lipoic Acid - Millipore Sigma, 437695-100UL, Lot #3030073
MCU - Cell Signaling Technology, 14997, Lot #1
MICU1 - Cell Signaling Technology, 12524, Lot #2
NDUFS1 - Cell Signaling Technology, 70264, Lot #1
NFS1 - Santa Cruz Biotechnology, sc-365308, Lot #H1617
NNT - Abcam, ab110352, Lot #GR3391830-1
NTHL1 - Proteintech, 14918-1-AP, Lot #00082606
PDH-E2 - Abcam, ab126224
POLD1 - Abcam, ab186407, Lot #1010142-3
PPAT - Proteintech, 15401-1-AP, Lot #00075223
PRDX3 - Abcam, ab73349, Lot #GR283600-1
SDHB - Cell Signaling Technology, 92649, Lot #1
SHMT2 - Cell Signaling Technology, 12762, Lot #1
TOM20 - Santa Cruz Biotechnology, sc-17764, Lot #G0212
alpha-Tubulin - Abcam, ab7291, Lot #GR3197113-7
Peroxidase conjugated goat anti-rabbit - Jackson Immunoresearch, Cat #111-035-003, Lot#127760.
Peroxidase conjugated goat anti-mouse - Jackson Immunoresearch, Cat #115-035-003, Lot#127655.

Validation

All antibodies used in this study are commercially available and have been validated by the manufacturer and in previous studies.

ACC - Cell Signaling Technology, 3662, https://www.cellsignal.com/products/primary-antibodies/acetyl-coa-carboxylase-antibody/3662
ACO1 - GeneTex, GTX128976, https://www.genetex.com/Product/Detail/Aconitase-1-antibody/GTX128976
ACO2 - GeneTex, GTX109736, https://www.genetex.com/Product/Detail/Aconitase-2-antibody-C1C3/GTX109736
AIF - Cell Signaling Technology, 4642, https://www.cellsignal.com/products/primary-antibodies/aif-antibody/4642?N=4294956287&Ntt=aif&fromPage=plp
ATF4 - Cell Signaling Technology, 11815, https://www.cellsignal.com/products/primary-antibodies/atf-4-d4b8-rabbit-mab/11815

Beta-Actin - Thermo Fisher Scientific, AM4302, https://www.thermofisher.com/antibody/product/beta-Actin-Antibody-clone-AC-15-Monoclonal/AM4302
Catalase - Cell Signaling Technology, 12980, https://www.cellsignal.com/products/primary-antibodies/catalase-d4p7b-xp-rabbit-mab/12980
CHAC1 - Proteintech, 15207-1-AP, https://www.ptglab.com/products/CHAC1-Antibody-15207-1-AP.htm
CHAC2 - GeneTex, GTX128819, https://www.genetex.com/Product/Detail/CHAC2-antibody/GTX128819
CS - Cell Signaling Technology, 14309, https://www.cellsignal.com/products/primary-antibodies/citrate-synthase-d7v8b-rabbit-mab/14309
DLST - Cell Signaling Technology, 11954, https://www.cellsignal.com/products/primary-antibodies/dlst-d22b1-xp-rabbit-mab/11954
DPYD - Cell Signaling Technology, 4654, https://www.cellsignal.com/products/primary-antibodies/dpyd-d35a8-rabbit-mab/4654
ERp44 - Cell Signaling Technology, 3798, https://www.cellsignal.com/products/primary-antibodies/erp44-d17a6-xp-rabbit-mab/3798
FECH - Santa Cruz Biotechnology, sc-377377, https://www.scbt.com/p/ferrochelatase-antibody-a-3
Grp94 - Cell Signaling Technology, 2104, https://www.cellsignal.com/products/primary-antibodies/grp94-antibody/2104
HSP60 - Cell Signaling Technology, 4870, https://www.cellsignal.com/products/primary-antibodies/hsp60-d307-antibody/4870
HSP90 - Cell Signaling Technology, 4874, https://www.cellsignal.com/products/primary-antibodies/hsp90-antibody/4874
ISCU - Santa Cruz Biotechnology, sc-373694, https://www.scbt.com/p/iscu1-2-antibody-d-6
Lamin B2 - Cell Signaling Technology, 12255, https://www.cellsignal.com/products/primary-antibodies/lamin-b2-d8p3u-rabbit-mab/12255
LIAS - Proteintech, 11577-1-AP, https://www.ptglab.com/products/LIAS-Antibody-11577-1-AP.htm
Lipoic Acid - Millipore Sigma, 437695-100UL, https://www.emdmillipore.com/US/en/product/Anti-Lipoic-Acid-Rabbit-pAb,EMD_BIO-437695
MCU - Cell Signaling Technology, 14997, https://www.cellsignal.com/products/primary-antibodies/mcu-d2z3b-rabbit-mab/14997
MICU1 - Cell Signaling Technology, 12524, https://www.cellsignal.com/products/primary-antibodies/cbara1-micu1-d4p8q-rabbit-mab/12524
NDUFS1 - Cell Signaling Technology, 70264, https://www.cellsignal.com/products/primary-antibodies/ndufs1-e4k3e-rabbit-mab/70264
NFS1 - Santa Cruz Biotechnology, sc-365308, https://www.scbt.com/p/nfs1-antibody-b-7
NNT - Abcam, ab110352, https://www.abcam.com/products/primary-antibodies/nnt-antibody-8b4bb10-ab110352.html
NTHL1 - Proteintech, 14918-1-AP, https://www.ptglab.com/products/NTHL1-Antibody-14918-1-AP.htm
PDH-E2 - Abcam, ab126224, https://www.abcam.com/products/primary-antibodies/pyruvate-dehydrogenase-e2-antibody-ab126224.html
POLD1 - Abcam, ab186407, https://www.abcam.com/products/primary-antibodies/pold1-antibody-epr15118-ab186407.html
PPAT - Proteintech, 15401-1-AP, https://www.ptglab.com/products/PPAT-Antibody-15401-1-AP.htm
PRDX3 - Abcam, ab73349, https://www.abcam.com/products/primary-antibodies/peroxiredoxin-3prdx3-antibody-ab73349.html
SDHB - Cell Signaling Technology, 92649, https://www.cellsignal.com/products/primary-antibodies/sdhb-e3h9z-xp-rabbit-mab/92649
SHMT2 - Cell Signaling Technology, 12762, https://www.cellsignal.com/product/productDetail.jsp?productId=12762
TOM20 - Santa Cruz Biotechnology, sc-17764, https://www.scbt.com/p/tom20-antibody-f-10
alpha-Tubulin - Abcam, ab7291, https://www.abcam.com/products/primary-antibodies/alpha-tubulin-antibody-dm1a-loading-control-ab7291.html
Peroxidase conjugated goat anti-rabbit - Jackson Immunoresearch, Cat #111-035-003, https://www.jacksonimmuno.com/catalog/products/111-035-003
Peroxidase conjugated goat anti-mouse - Jackson Immunoresearch, Cat #115-035-003, https://www.jacksonimmuno.com/catalog/products/115-035-003

Eukaryotic cell lines

Policy information about cell lines and Sex and Gender in Research

Cell line source(s)	H322, H1792, H1975, and PC9 NSCLC cell lines were obtained from the Harmon Cancer Center Collection (University of Texas-Southwestern Medical Center). Phoenix-Ampho 293T cells and A549, H1299, and H2009 NSCLC cells were obtained from the American Type Culture Collection (ATCC). Lenti-X-293T cells were obtained from Clontech.
Authentication	All cell lines were authenticated by Short Tandem Repeat (STR) profiling by the provider.
Mycoplasma contamination	All cell lines were routinely tested for mycoplasma and confirmed to be mycoplasma free.
Commonly misidentified lines (See ICLAC register)	No commonly misidentified cell lines were used in this study.

Flow Cytometry

Plots

Confirm that:

- [ ] The axis labels state the marker and fluorochrome used (e.g. CD4-FITC).
- [ ] The axis scales are clearly visible. Include numbers along axes only for bottom left plot of group (a 'group' is an analysis of identical markers).
- [ ] All plots are contour plots with outliers or pseudocolor plots.
- [ ] A numerical value for number of cells or percentage (with statistics) is provided.

Methodology

Sample preparation	For all analyses, cells were washed twice with ice cold PBS following incubation with the respective fluorescent probe and collected into 0.5mL of ice cold PBS for analysis.
Instrument	A BD Accuri C6 Plus Flow Cytometer (BD Biosciences, Franklin Lakes, NJ, USA) was used for analyses of ROS.
Software	BD CSampler Plus software (BD Biosciences, Franklin Lakes, NJ, USA) was used to collect raw flow cytometry data and GraphPad Prism 9.4.1 (458) was used for analysis of the mean fluorescence intensity of positively stained cells.
Cell population abundance	No sorting was performed for the described analyses.
Gating strategy	For all analyses, live NSCLC cells were identified with an initial FSC/SSC gating scheme (FSC > 500,000; SSC > 500,000). Cell doublets were then excluded through subsequent gating based on forward scatter-height and forward scatter-area (FSC-H v. FSC-A). Cells positively stained for the respective fluorescent ROS indicator were identified based on gating established by determining the FITC and/or PE autofluorescence of unstained control cells. Cells present to the left of these gates on the corresponding histograms were considered unstained, where those to the right of these gates were considered positively stained and included for analysis.

☒ Tick this box to confirm that a figure exemplifying the gating strategy is provided in the Supplementary Information.
